# Self-reported quality of life in progressive cognitive decline—let’s not throw the baby out with the bathwater

**DOI:** 10.1007/s11136-025-04009-7

**Published:** 2025-06-27

**Authors:** Irina Kinchin

**Affiliations:** Centre for Health Policy and Management, School of Medicine, Trinity College Dublin, Dublin, Dublin 2 Foster Pl Ireland

**Keywords:** Dementia, Progressive conditions, Quality of life, Assessment, Health economics, Proxy reporting, Cognitive justice, Methodological innovation

## Abstract

Assessing quality of life in progressive conditions like dementia presents unique challenges for Health Economics and Outcomes Research (HEOR). This commentary discusses current practices, highlighted by recent appraisals of lecanemab by NICE, where concerns about proxy-reported data—specifically, the risk of underestimating benefits—were noted as a limitation in the evaluation. While progressive cognitive decline complicates traditional self-reporting methodologies, emerging evidence demonstrates that adaptive strategies and technology-assisted methods can extend reliable self-reporting windows. The analysis identifies four critical challenges: (1) cognitive heterogeneity across dementia subtypes and stages clashing with HEOR's stable preference assumptions; (2) temporal fluctuations undermining single-timepoint assessments; (3) systematic discrepancies between self- and proxy-reported outcome data, inadequately addressed by current methods; and (4) the conceptual and psychometric limitations of current generic and condition-specific instruments. To address these challenges, the author advocates for a paradigm shift, inviting HEOR community to view diverse expressions of preference as a catalyst for innovation. This involves moving beyond static, binary preference assumptions to embrace dynamic, multimodal methods capturing non-verbal expressions in more advanced dementia. Proposed solutions include proxy calibration, an ecosystem of hybrid and graduated assessment integrating standardized domains with adaptable modules; reforming proxy protocols using Pickard's dual-gap framework to position proxy input as complementary, not substitutive; employing modern psychometrics and adopting relationship-centred engagement and process consent. Crucially, it calls for HEOR to integrate adaptive methods from adjacent fields—such as phenomenological inquiry, sensor technology, and data triangulation. These approaches can enhance the ability of HEOR to measure, value, and assess quality of life in the context of dementia and other progressive neurodegenerative conditions, ultimately anchoring value in lived experience and ensuring methodological limitations do not become barriers to accessing care and treatments.

## Introduction

Quality of life measurement and valuation in health economics and outcomes research (HEOR) defaults to proxy reporting at diagnosis of dementia in conditions such as Lewy body dementia, thus devaluing the person's own perspective and lived experience. Extensive evidence, including a 2024 systematic review and meta-analysis of 96 studies (635 observations) [1], draw attention to systematic differences between self-reported and proxy-reported quality of life in dementia research [2]. This premature shift to proxy assessment risks generating incomplete or biased evidence about the experiences and preferences of people living with dementia, which can adversely affect care and treatment decisions.

A question arises: Could HEOR methodologies for quality of life measurement and valuation benefit from more adaptive strategies that evolve alongside cognitive trajectories? Emerging evidence from the ARMADA study shows promising possibilities, with certain computerized instruments maintaining reliability even as cognitive abilities fluctuate [3]. These findings suggest that quality of life assessment in HEOR does not need to default to proxy reporting at the first sign of cognitive impairment, indicating that technology-assisted tools could potentially extend the window of reliable self-reporting in progressive neurodegenerative conditions, while also creating opportunities for proxy calibration during periods of overlapping capacity. This commentary argues how HEOR methodologies for quality of life assessment can be advanced as a continuum rather than a binary (valid/invalid) classification, proposing an 'ecosystem of values', where self-reported experiences, proxy observations, physiological indicators, and behavioural expressions interact to create a more complete understanding of quality of life across cognitive trajectories. Yet translating these technological possibilities into practice requires broader systemic changes.

Implementation science further invites us to consider whether inclusive methods might actually enhance the precision of intervention targeting, despite requiring additional resources [4]. Furthermore, we should consider the relationship between researcher communication competence and research quality. Could specialised communication training for researchers improve both data quality and participant engagement in dementia research? These foundational considerations might help reconcile the tension between methodological rigor and ethical inclusion, particularly when distinguishing between the roles of PPI (Patient and Public Involvement) advisors (who support the shaping research design) and research participants (from whom data are derived). Early evidence suggests these goals might be mutually reinforcing, not competing [5]. However, the urgency of addressing these methodological limitations extends beyond academic debate.

The stakes of these methodological failures have never been higher. NICE's repeated rejections of lecanemab in 2024 represent a historic inflection point [6]. After decades of symptomatic treatments that offered temporary relief without addressing underlying disease progression, lecanemab emerged as the first disease-modifying treatment for Alzheimer's disease to target the root pathological processes. Yet crucially for HEOR methodology, NICE explicitly cited concerns that "utility values may have been underestimated by using patient-by-proxy reported quality of life data". This concern reveales a profound paradox: breakthrough treatments that could slow cognitive decline are being denied to patients partly because our measurement tools undervalue their lived experience. When proxy assessments become gatekeepers to innovation, methodological conservatism transforms into a barrier to medical progress. The question is no longer whether HEOR can afford to evolve its measurement approaches for people living with dementia—it's whether we can afford not to. Every delayed methodological innovation potentially delays access to treatments that could preserve the very cognitive abilities our current measurement tools struggle to capture.

### The complexity of lived experience

Dementia is an incurable, degenerative, and disabling condition that originates neurologically but unfolds within a social context [7]. Currently, more than 55 million people live with dementia worldwide, a number projected to nearly triple to 139 million by 2050 [8]. This growing prevalence has significant implications, as the progressive nature of dementia extends far beyond its neurological origins and introduces profound uncertainties that ripple through individual lives, relationships, and healthcare systems.

Each person's journey navigating through the clinically defined stages of mild, moderate, and advanced dementia is uniquely personal, complicated further by the heterogeneous nature of cognitive changes and distinct manifestations across different types of dementia—from the memory impairment and cortical atrophy in Alzheimer's disease, executive dysfunction and gait disturbances in vascular dementia, to fluctuating cognition and recurrent visual hallucinations characteristic of Lewy body dementia and behavioural disinhibition in behavioural variant frontotemporal dementia or language difficulties in primary progressive aphasia, among other subtypes [9]. It is essential to recognize that dementia serves as an umbrella term encompassing various neurodegenerative conditions, rather than being synonymous with Alzheimer's disease as is commonly misunderstood [10]. While Alzheimer's is the most prevalent form—accounting for 60–70% of cases [11]—understanding the full spectrum of dementia is essential for equitable HEOR, from burden of illness studies to the assessment of intervention effectiveness, that ultimately inform diagnosis and care.

As the seventh leading cause of death globally and a significant cause of disability and dependency among older adults, dementia represents one of the most pressing health and social care challenges of the twenty-first century [11]. Its multifaceted nature defies traditional HEOR methodologies at every level, from participant recruitment and data collection to analysis and decision-making.

### From exclusion to empowerment

Historically, research has approached dementia through a positivist biomedical lens, often excluding those living with the condition from active participation, as considered incapable of contributing meaningfully to the understanding of their condition [12, 13]. This exclusion was partly due to practical and methodological challenges, compounded by an ‘ethical paternalism’ that deemed individuals with dementia as especially vulnerable and in need of protection [14].

Recent years have witnessed a shift toward greater societal engagement with people living with dementia, emphasizing advocacy and increased participation in research — from development to dissemination [15, 16]. Yet, HEOR methodologies for quality of life assessment have been slow to evolve, often defaulting to proxy reports. Although valuable, relying prematurely or exclusively on proxy reports risks obscuring the direct experiences of people with dementia, as proxy perceptions often differ from the individual's [1]. This misalignment can lead to care unresponsive to personal preferences and perpetuate marginalization by silencing their voice [17].

In drawing parallels between the voices of children and those of people with dementia, the fundamental recognition is that all individuals, regardless of age or cognitive ability, are embedded within a web of social relations, healthcare systems, and community support structures that significantly influence how they express themselves and make sense of the world around them. Understanding these expressions requires a committed, interpretive effort to see the world as they see it, acknowledging the fullness of their voices as part of the human community [18].

### A call for methodological innovation in HEOR

Building on this imperative, this commentary advocates for a paradigm shift in how HEOR approaches quality of life assessment within dementia research and progressive neurodegenerative conditions more broadly. By adopting HEOR methodologies that honour diverse ways of expressing preferences and value individual agency—focusing on expressed abilities rather than deficits, one would argue, we can foster more equitable and sustainable healthcare systems.

Growing paediatric (or child) HEOR has demonstrated that meaningful assessment is possible through age-appropriate measures and adaptive methodologies [19]. If we develop effective HEOR methods to assess quality of life in children with developing cognitive abilities, we should be able to apply similar thinking to create appropriate assessment for people living with dementia. The key difference is direction —while paediatric assessment tracks developing capabilities, dementia assessment must accommodate progressive decline, requiring distinct but equally thoughtful approaches.

The following sections discuss several methodological challenges in current HEOR practice and potential directions for evolving the research landscape.

#### Challenge 1: Cognitive heterogeneity across dementia subtypes and stages

HEOR methodologies for quality of life assessment face inherent tensions when applied to neurodegeneration, rooted in the discipline’s foundational assumption of stable preferences and cognitive consistency [20–22]. For example, traditional quality of life valuation tools such as discrete choice experiments (DCEs), time trade-off (TTO) tasks, and other preference elicitation methods used to derive quality-adjusted life-years (QALYs) rely on rational choice frameworks and cognitive capabilities that are often compromised by the progressive neurocognitive heterogeneity of dementia. This mismatch is particularly evident across different subtypes and stages of the condition.

Alzheimer’s disease, characterized by progressive memory impairment, challenges recall-dependent methods like retrospective quality of life assessments and preference elicitation tasks requiring stable autobiographical memory. Similarly, frontotemporal dementia, associated with executive dysfunction and behavioural changes, limits the validity of tools that require complex decision-making or consistent judgment, such as DCEs and TTO tasks. Furthermore, Lewy body dementia, with its fluctuating attention and awareness alongside visuospatial difficulties, can impede consistent engagement during assessment, affecting the reliability of responses across different timepoints.

Even within the same dementia subtype, progression through different stages introduces unique methodological challenges. In the early stages (including mild cognitive impairment and mild dementia), adapting standard HEOR methods like DCEs or TTO tasks might be feasible with appropriate support. However, as the condition progresses into moderate stages, cognitive fluctuations often necessitate more flexible, adaptive, or hybrid assessment strategies combining self-report with observational data. In advanced dementia, reliance typically shifts towards observational methods or carefully considered proxy-supported approaches, acknowledging the complexities of proxy reporting discussed later. This wide spectrum highlights the need for methodologies tailored not just to the dementia subtype but critically also to the disease stage. For example, a DCE validated for mild Alzheimer’s disease might be unsuitable for someone with moderate frontotemporal dementia due to executive function demands, potentially leading to selection bias (exclusion) or yielding unreliable data if completed under significant cognitive strain. This mismatch between static assessment tools and dynamic disease progression shows why HEOR methodologies must evolve beyond one-size-fits-all approaches toward more adaptive, stage-sensitive frameworks that can capture valid and meaningful preferences across the full spectrum of cognitive change.

#### Challenge 2: Temporal fluctuations in cognitive function 

Beyond these stage-specific challenges, people living with dementia experience significant intra-individual cognitive variability, which includes both short-term fluctuations (“good days/bad days”) and longer-term neurodegenerative progression. This temporal dynamic challenges the reliability of cross-sectional HEOR data and the assumption of stable health states [23]. For example, in Lewy body dementia, attention and awareness can fluctuate dramatically within hours, making single-timepoint assessments potentially unrepresentative of typical experiences and challenging the validity of derived utility values. Similarly, longitudinal studies face difficulties, as participants with rapidly progressing subtypes, such as behavioural variant frontotemporal dementia, may lose the capacity to complete complex preference tasks (e.g., standard gamble) over the study period, leading to informative censoring.

This temporal variability has measurable consequences for HEOR instruments. The EQ-5D, commonly used in HEOR to estimate QALYs and inform cost-effectiveness analyses and healthcare resource allocation [24], exemplify this challenge. Studies have demonstrated poor test–retest reliability for the EQ-5D-5L in moderate-to-severe dementia, characterized by low intraclass correlation coefficients (ICCs) and significant variability [25, 26]. With ICCs falling below the 0.6 threshold often considered acceptable [27], these findings strongly suggest that traditional assumptions of stable preferences underpinning many HEOR valuations may not hold, particularly as dementia progresses. While dementia-specific instruments like DEMQOL or QOL-AD aim to improve content relevance [28, 29], the inherent cognitive fluctuations still pose challenges to achieving stable psychometric properties like test–retest reliability and responsiveness across all disease stages.

Methods like smartphone-based Ecological Momentary Assessment (EMA), along with strategies such as scheduling shorter assessments or timing them during periods of optimal cognitive function, offer potential pathways to capture real-time preferences and achieve a more nuanced understanding of this temporal variability [30, 31]. However, implementing these approaches is not without challenges. Limitations include potential participant burden (especially with significant cognitive or motor impairments), the possible need for proxy input within EMA frameworks if self-report becomes infeasible, and practical concerns around accessibility, usability, and data security. Therefore, adapting these methods—or developing novel ones—to be truly feasible, acceptable, and sensitive to the dynamic nature of lived experience in dementia remains a critical area for methodological advancement in HEOR.

#### Challenge 3: Proxy-person differences

As previously noted, extensive evidence draws attention to systematic differences between self-reported and proxy-reported quality of life in dementia research. The choice of proxy perspective critically influences outcomes, echoing Pickard & Knight's seminal framework [32] which distinguishes between proxy-person perspectives (attempting to simulate the person's viewpoint) and proxy-proxy perspectives (providing the proxy's own observations). This dichotomy creates both an inter-rater gap (divergence between self-reports and proxy-person attempts) and an intra-proxy gap (difference between proxy-person simulations and proxy-proxy observations made by the same proxy). A meta-analysis reveals these gaps persist in dementia care [1], with proxy-proxy perspectives (Standardized Mean Difference [SMD] = 0.532) showing substantially greater divergence from self-reports than proxy-person attempts (SMD = 0.250). This suggests that achieving accurate perspective-taking may be particularly challenging for caregivers in the dementia context. This pattern was observed across multiple quality of life instruments, including the QOL-AD, EQ-5D, and DEMQOL, though heterogeneity remained high due to variability in study designs and populations [1].

While originally developed in oncology, Pickard's framework [32] proves salient in dementia, where progressive cognitive decline can amplify both inter-rater and intra-proxy gaps. By explicitly acknowledging these dual gaps, researchers can design proxy protocols that aim to separately capture caregiver observations (proxy-proxy) and perspective-taking attempts (proxy-person), rather than conflating these distinct viewpoints, thereby potentially improving the clarity and utility of proxy data.

Examining different types of caregivers reveals further nuances. Family caregivers adopting the proxy-proxy perspective often show the largest discrepancies compared to self-reports, potentially anchoring assessments to the person’s pre-dementia state rather than their current condition [1]. Formal caregivers (e.g., care staff), who may focus more on observable symptoms or compare individuals to others in clinical settings, tend to report smaller gaps, possibly reflecting greater clinical detachment [33]. Dementia severity further modulates these differences: proxy-proxy divergences tend to widen as cognitive impairment progresses, whereas proxy-person ratings often align more closely with self-reports in mild-to-moderate stages [34, 35].

While overall agreement between self- and proxy-reports in dementia is often low (pooled Intraclass Correlation Coefficient [ICC] ≈ 0.30 reported in the meta-analysis), proxy-person ratings showed marginally better concordance (ICC ≈ 0.36) than proxy-proxy assessments (ICC ≈ 0.26). This highlights the potential, albeit limited, role of the proxy-person perspective in approximating subjective quality of life when direct reporting becomes unreliable or impossible.

Dementia-specific tools like DEMQOL, with their dual self-report (DEMQOL) and proxy-report (DEMQOL-Proxy) formats, represent attempts to formally address this challenge. However, even these tools face limitations; the self-report version relies on verbal responsiveness and interviewer administration, becoming less feasible in severe dementia (where the developers recommend using only DEMQOL-Proxy), potentially excluding individuals expressing preferences through non-verbal means. Nonetheless, when caregivers provide their own observational assessments (proxy-proxy ratings), these remain valuable for capturing distinct observational insights (reflecting the intra-proxy gap), providing potentially unique and clinically relevant information, as argued by Pickard & Knight [32].

A natural extension of Pickard's framework might involve 'proxy calibration windows'—structured periods where caregivers learn to interpret preferences during overlapping self-report and proxy capacity. Realizing this potential would require reimagining proxy protocols as dynamic, educative processes where caregivers transition from passive observers to trained interpreters of lived experience, creating a promising direction for narrowing the inter-rater gap over time. By "trained observers of lived experience," this envisions caregivers developing skills in: (1) recognize subtle indicators of subjective experiences (such as comfort levels, preferences, and emotional states) beyond merely observable behaviours; (2) distinguishing between their own interpretations and the person's actual lived experience; and (3) applying both proxy-person (what the person would report) and proxy-proxy (caregiver's own assessment) perspectives as complementary rather than competing viewpoints. This approach could transform proxy assessment from static reporting into skilled interpretation, where caregivers navigate multiple perspectives to provide more nuanced assessments.

#### Challenge 4: Psychometric limitations of HEOR instruments

Preference-based instruments commonly used in HEOR, such as the EQ-5D, Short Form-36 (SF-36), or Health Utilities Index (HUI), serve as foundational tools for assessing a health status or health-related quality of life (HRQoL) and informing economic evaluations. However, these generic quality of life tools often apply standardized frameworks that may not fully capture the unique dimensions and complexities of quality of life as experienced by people living with dementia [36, 37].

The dominant conceptual framework underpinning many generic HEOR instruments assumes a universal hierarchy of health domains (e.g., mobility, pain, self-care) that can be uniformly weighted and aggregated into a single utility score. This approach aligns well with classical biomedical models prioritizing physical functioning and symptom reduction. Yet, this perspective contrasts sharply with more holistic frameworks. For example, the model by Wilson and Cleary emphasizes the interplay between biological, psychological, and social determinants of health outcomes [38]. Similarly, the World Health Organization's International Classification of Functioning, Disability and Health (ICF) framework distinguishes between body impairments, activity limitations, and participation restrictions, incorporating contextual factors like social attitudes and physical environments [39]. Ferrans et al. proposed integrating personal preferences and cultural values [40], while Huber et al. emphasizes resilience and autonomy [41]. Engel's biopsychosocial model expands the scope beyond physical health to include psychological resilience and social connectedness, with systems like PROMIS® reflecting similar multidimensional approaches [42].

Recognizing these gaps, dementia-specific instruments like the DEMQOL, QOL-AD were developed to better align with lived experience. DEMQOL, for example, was developed using qualitative input from people living with dementia and their carers to establish a conceptual framework encompassing domains like daily activities, health and well-being, cognitive functioning, social relationships, and self-concept [28]. However, even these instruments face the fundamental challenge of operationalizing complex, fluid constructs within standardized psychometric formats. The development process for DEMQOL highlighted challenges in establishing stable subscales reflecting its initial conceptual framework and noted difficulties in assessing responsiveness, a common issue for dementia HRQoL measures. Furthermore, while DEMQOL's interviewer-administered format aims to reduce cognitive burden, its reliance on verbal responsiveness limits the feasibility of self-report in severe dementia (MMSE < 10), where the developers recommend using only the proxy version (DEMQOL-Proxy). This illustrates a broader pattern where even well-intentioned, dementia-specific instruments struggle to bridge conceptual ambitions with practical measurement realities.

The disconnect between richer conceptualizations and standard HEOR instruments leads to tangible psychometric consequences. The assumption of universal domain relevance undermines content validity, particularly in diverse cultural contexts, as shown by Jelsma and Ferguson in South Africa regarding the SF-36 [43]. Generic tools like EQ-5D and HUI also lack the granularity to measure psychosocial subtleties crucial in many conditions, such as stigma or social functioning in depression [44].

Methodologically, addressing these limitations requires moving beyond classical test theory. Modern psychometric approaches like Item Response Theory (IRT) and computerized adaptive testing (CAT), used in PROMIS®, may offer tailored and less burdensome assessments [45]. Hybrid models combining core generic domains with modular, condition-specific or culturally adaptable add-ons—akin to the WHOQOL-BREF's structure [46]—represent one promising direction. Furthermore, economic evaluations could benefit from integrating complementary evaluative frameworks, such as the capability approach [47–49], which prioritizes individuals' freedom to achieve wellbeing using measures like the ICECAP instruments for older people (ICECAP-O) and adults (ICECAP-A) that assess broader dimensions like attachment, security, role fulfillment, enjoyment, and control [50–52]. 

.

### Vision for the future research: from proxy to personhood

#### The epistemological challenge

This commentary invites the HEOR community to reconceptualize dementia—not merely as a monolithic clinical entity, but as a constellation of diverse lived experiences shaped by cognitive trajectories, social contexts, and individual narratives. At the heart of this reconceptualization lies a critical question: What if HEOR's foundational assumption—that all health states can be adequately measured through stable, cognitively articulated preferences—proves not only impractical for progressive neurodegeneration but ethically untenable?

Current HEOR methodologies for quality of life assessment expose a concerning inconsistency. While often prematurely defaulting to proxy assessments even when limited self-reporting remains possible, HEOR simultaneously prioritizes individuals capable of "rational" self-reporting—a dual exclusion that mirrors disability scholars' concept of methodological ableism. This inherent bias disadvantages those expressing preferences through non-normative cognitive channels, effectively silencing embodied, emotional, and relational forms of communication.

To counter this epistemic injustice, what transformative shifts might we consider in HEOR? Perhaps expanding preference elicitation by redefining "preference" to encompass dynamic, non-verbal expressions of wellbeing could help bridge the cognitive diversity gap. In advanced dementia, could HEOR frameworks integrate analyses of gestural communication patterns, physiological markers such as cortisol responses during meaningful activities (e.g., music therapy), or biometric indicators like heart rate variability captured during care interactions [53]? This expanded 'ecosystem of values' would draw from methods more common in clinical observation or psychosocial research, creating pathways for HEOR to value how individuals express quality of life on their own terms, rather than filtering experiences through conventional communication hierarchies ill-suited to advanced dementia.

It is crucial to acknowledge that many approaches discussed here —such as analysing non-verbal communication, employing phenomenological inquiry, or prioritizing relationship-centred engagement—draw from disciplines like social sciences, nursing research, qualitative methodologies. Indeed, some are already utilized effectively in dementia care practice and research outside the specific remit of HEOR. However, the vision here is not simply to import these methods wholesale, but rather for HEOR to integrate and adapt them. The goal is to leverage these insights specifically to enhance the validity and ethical grounding of HEOR's core tasks—measurement, valuation, and economic evaluation for informing decision-making — when faced with the profound challenges of progressive cognitive decline, challenges that standard HEOR tools struggle to meet alone. This integration aims to generate more robust inputs for economic models and ultimately inform more person-centred resource allocation, rather than replacing HEOR's distinct evaluative function.

What if we reformed proxy protocols by explicitly building on Pickard's dual-gap framework [32]? This could underpin proxy calibration windows and caregiver training designed to develop perspective-taking skills, thereby narrowing the inter-rater gap, while also capturing distinct observational insights relevant to the intra-proxy gap. Might this foster a multidimensional assessment model that honours both lived experience and clinical observation across disease stages?

Could institutionalizing cognitive justice—framing communication diversity as a catalyst for methodological innovation rather than a measurement obstacle—allow HEOR to develop an ecosystem of graduated assessment systems? Such systems might prioritize preserving autonomy through adapted self-reporting tools, validate non-verbal expression as legitimate preference data, and strategically position proxy inputs as complementary, rather than substitutive, to the person's own expressions whenever possible.

#### Reimagining HEOR engagement

What might HEOR engagement look like when we acknowledge the distinct yet complementary roles people with dementia already fulfill in research—balancing their advisory contributions in research design (Patient and Public Involvement, PPI) with participatory adaptations in data collection? As Gove et al. [54] note in their position paper for Alzheimer Europe, establishing trust through repeated interactions and adapted communication methods (e.g., visual aids, simplified language) creates space for people living with dementia to shape research priorities and protocols as active collaborators, enhancing the relevance of HEOR studies.

When shifting focus to involving people with dementia as participants providing data for HEOR studies, methodological adaptations become essential. Could cognitive assessments like the Montreal Cognitive Assessment (MoCA) serve not as exclusionary gatekeepers but as guides for tailoring HEOR methods? For example, DCEs might remain feasible for individuals with mild cognitive impairment, while observational, biometric, or sensor-based approaches could better capture preferences and experiences in more advanced dementia. Environmental considerations, such as providing quiet settings to reduce sensory overload and adapting assessment windows (e.g., 20 min) to accommodate cognitive fatigue, should be treated as integral scientific variables impacting data quality, rather than mere logistical afterthoughts [55].

Just as environmental adaptations can mitigate sensory overload, relational adaptations are crucial for addressing fluctuating cognitive capacities. McKeown et al. [56] have shown that relationship-centred approaches—treating rapport-building as a methodological imperative rather than procedural courtesy—can lead to improved engagement and potentially higher data quality. Similarly, the concept of "process consent," rooted in ethical practice and qualitative research, reimagines consent not as a one-time signature but as an iterative exchange [57]. This involves ongoing dialogue and adaptive decision-making to accommodate fluctuating capacity. While originating outside traditional HEOR protocols, adopting such relational and ethical practices is arguably essential for HEOR to maintain validity and legitimacy when engaging participants whose cognitive capacity fluctuates or diminishes.

Both dimensions of engagement—valuing people with dementia as expert advisors (PPI) and adapting methods for their role as research participants—demand attention if HEOR is to evolve beyond proxy assumptions toward authentic, person-centred assessment of value based on the lived experience of dementia.

#### Interpreting diverse expressions: from subjective experience to care metrics

To preserve personhood, approaches to assessing quality of life must evolve alongside cognitive trajectories, potentially through graduated methodologies that respect changing communication needs [36]. First, adapting traditional tools could offer accessibility for those with mild cognitive changes. Scales enhanced with pictorial anchors, for instance, might preserve direct self-reporting while reducing cognitive load; early research suggests such adaptations can maintain reliability while increasing engagement compared to text-heavy alternatives [58]. Phenomenological approaches, while traditionally the domain of qualitative inquiry, can provide invaluable insights for HEOR by identifying what truly matters to individuals [59–63], thereby informing the selection of relevant attributes for preference elicitation or item selection for quality of life measures. For example, qualitative insights into the value of sensory pleasures (e.g., enjoying sunlight) could shape DCE attributes for individuals with semantic dementia, whose lexical impairments render traditional surveys inadequate. When verbal expression becomes limited, observational methods—often used in nursing or behavioural science—can help HEOR to capture data that would otherwise be lost.

Hybrid methods offer a powerful way to bridge verbal and non-verbal expression, potentially extending existing generic or dementia-specific tools by integrating their domains with other data sources. For example, DEMQOL's items related to subjective experience (e.g., 'enjoying life') or relational aspects (e.g., 'treatment by others') could be enriched. Real-time physiological data, such as galvanic skin response measured during meaningful activities like music therapy sessions, could provide objective markers of engagement or emotional state. Simultaneously, narrative prompts using 'guided storytelling' could elicit subjective experiences indirectly when direct questioning is difficult. Combining these methods creates an ecosystem of layered assessments that honour both structured reports (self or proxy) and embodied expressions of well-being, potentially revealing quality of life indicators when explicit articulation becomes challenging. Furthermore, coding nonverbal behaviours (e.g., eye contact duration during interactions, responses to stimuli) or using tools like "Talking Mats" (visual symbol-based communication aids), developed in communication therapy, offer practical methods adaptable for HEOR to decode preferences that transcend language in later-stage dementia [53].

More collaborative approaches might also honour remaining communication while acknowledging increasing support needs. Dyadic interview techniques or group decision-making, where care partners help contextualize observed behaviours or assist with communication without supplanting the person's own expressions, could offer a middle path between complete autonomy and proxy substitution, particularly when used during calibration periods to enhance proxy accuracy for later stages.

Data triangulation—combining diverse data sources such as adapted self-reports, proxy reports (both proxy-person and proxy-proxy perspectives), direct behavioural observations, and sensor-based physiological metrics—offers a crucial strategy for mitigating the systematic biases inherent in relying on any single method, especially proxy assumptions. For example, wearable devices tracking physiological responses (like heart rate variability or electrodermal activity) during specific activities can potentially validate or challenge caregiver-reported quality of life scores related to those activities, reducing over-reliance on second-hand interpretations. Such triangulation helps ensure that preferences inferred from non-verbal cues (e.g., signs of comfort or distress in advanced dementia) are considered alongside, and potentially used to refine interpretations of, verbal or proxy-derived data. This approach could be particularly valuable during proxy calibration windows, where multiple data sources help train caregivers to interpret preferences more accurately. Further research is essential to establish the reliability, validity, and optimal integration strategies for calibration approaches across different stages and types of cognitive impairment.

Bridging from improved engagement and measurement to data analysis and decision-making, economic evaluations must also adapt to better reflect the complexities of dementia. They could strengthen policy relevance by explicitly modelling the uncertainty inherent in diverse preference data derived from varied sources and fluctuating cognitive states, perhaps by weighting sensitivity analyses according to cognitive trajectory subgroups or perceived data source reliability. Generating robust inputs for such nuanced evaluations requires suitable elicitation methods; here, the Analytic Hierarchy Process (AHP) shows early promise. Mohr et al. [64] demonstrated its feasibility using simplified pairwise comparisons suitable for some individuals with cognitive impairment, although broader validation remains critical. Ultimately, implementing this suite of varied approaches—from the adapted measurement, engagement, elicitation, and evaluation techniques discussed throughout this commentary—could foster more ethically informed and person-centred resource allocation. This could help shift resources away from interventions like antipsychotics (which carry significant risks despite limited efficacy [65]) towards accessible options like music therapy and sensory-based approaches that show promise in supporting wellbeing at potentially lower cost [66, 67]. Crucially, these methodological innovations could help ensure that future treatment decisions are informed by more accurate representations of lived experience, avoiding the proxy-related concerns that initially complicated NICE's lecanemab evaluation.

## Concluding remark

This commentary argues that dementia does not obscure personhood; instead, it discusses the limitations of quality of life metrics and processes in HEOR that conflate cognitive function with human value. By embracing diverse ways of expressing preferences as a catalyst for innovation, HEOR can forge more ethically grounded and person-centred approaches to measurement, valuation, and decision-making.

Consider how different dementia types might reshape our understanding of quality of life in HEOR: people with Alzheimer's disease may find meaning in emotional connections in the present moment even as autobiographical memory and the ability to plan for the future fade, challenging utility frameworks that privilege future planning. Those with Lewy body dementia might experience heightened sensory appreciation during lucid periods, revealing the inadequacy of static measurement approaches. Frontotemporal dementia's disinhibition can expose authentic preferences typically masked by social conformity, offering insights into preference formation that could change choice modelling. Even advanced vascular dementia, where communication may be severely limited, demands methodological creativity—perhaps through physiological markers of comfort that capture quality of life beyond verbal articulation.

However, translating these insights into practice presents formidable challenges. Developing new measurement approaches requires extensive validation studies, substantial funding, and interdisciplinary collaboration that may strain existing research infrastructures. Training caregivers as skilled interpreters demands time and resources that healthcare systems may struggle to provide. Regulatory bodies and payers may resist methodological innovations that complicate established evaluation frameworks. The path forward demands not only a paradigm shift in thinking—measuring with humility, listening with intentionality, and anchoring value in lived experience rather than statistical expediency—but also sustained commitment to overcoming these practical barriers. This involves HEOR learning from and integrating insights from adjacent disciplines, acknowledging that such integration will be neither swift nor straightforward. Finally, implementing these diverse methodological innovations in isolation may limit their impact. Realizing their potential requires moving beyond piecemeal adoption toward an integrated ‘ecosystem of values’. The true progress begins when we accept both the imperative and the difficulty of moving beyond measuring what is easily quantifiable toward valuing what truly matters. 

## Data Availability

There is no data with this manuscript

## Declarations

### Précis

Current HEOR methodologies for assessing quality of life in progressive conditions such as dementia need adaptive, person-centred methods valuing cognitive diversity, non-verbal cues, and interdisciplinary insights for ethical, valid results.
